# Draft genome of *Bugula neritina*, a colonial animal packing powerful symbionts and potential medicines

**DOI:** 10.1038/s41597-020-00684-y

**Published:** 2020-10-20

**Authors:** Mikhail Rayko, Aleksey Komissarov, Jason C. Kwan, Grace Lim-Fong, Adelaide C. Rhodes, Sergey Kliver, Polina Kuchur, Stephen J. O’Brien, Jose V. Lopez

**Affiliations:** 1 Center for Algorithmic Biotechnology, Institute of Translational Biomedicine, St. Petersburg State University, St. Petersburg, 199034 Russia; 2 Applied Genomics Laboratory, SCAMT Institute, ITMO University, Saint Petersburg, 197101 Russia; 3 Division of Pharmaceutical Sciences, School of Pharmacy, University of Wisconsin-Madison, Madison, WI 53706 USA; 4 Department of Biology, Randolph-Macon College, Ashland, VA 23005 USA; 5 Zoologistics Consulting, Salem, MA 01970 USA; 6 Institute of Molecular and Cellular Biology, Siberian Branch of the Russian Academy of Sciences, Novosibirsk, 630090 Russia; 7 Genomic Diversity Laboratory, ITMO University, Saint Petersburg, 197101 Russia; 8 Halmos College of Arts and Sciences, Nova Southeastern University, Ft Lauderdale, FL 33314 USA

**Keywords:** Evolution, Computational biology and bioinformatics

## Abstract

Many animal phyla have no representatives within the catalog of whole metazoan genome sequences. This dataset fills in one gap in the genome knowledge of animal phyla with a draft genome of Bugula neritina (phylum Bryozoa). Interest in this species spans ecology and biomedical sciences because *B. neritina* is the natural source of bioactive compounds called bryostatins. Here we present a draft assembly of the *B. neritina* genome obtained from PacBio and Illumina HiSeq data, as well as genes and proteins predicted de novo and verified using transcriptome data, along with the functional annotation. These sequences will permit a better understanding of host-symbiont interactions at the genomic level, and also contribute additional phylogenomic markers to evaluate Lophophorate or Lophotrochozoa phylogenetic relationships. The effort also fits well with plans to ultimately sequence all orders of the Metazoa.

## Abstract (summary)

**Table:** 

Measurement(s)	DNA • genome • sequence_assembly • sequence feature annotation
Technology Type(s)	DNA sequencing • sequence assembly process • sequence annotation
Sample Characteristic - Organism	Bugula neritina

Machine-accessible metadata file describing the reported data: 10.6084/m9.figshare.12988355

## Background & Summary

**Fig. 1:**
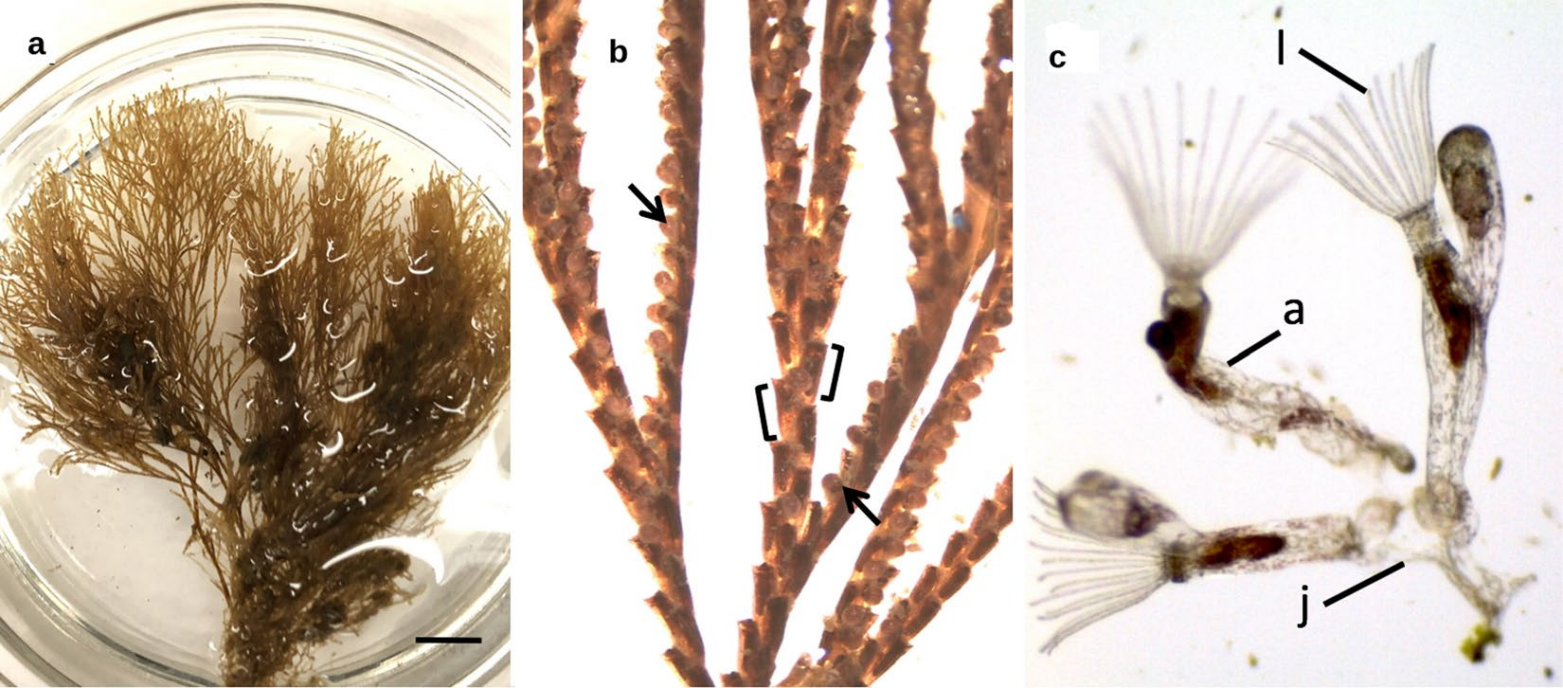
(**a**) Whole colony of a preserved *B. neritina*. Scale bar represents 10 mm. (**b**) Light micrograph of a preserved fecund *B. neritina* colony, with feeding zooids (in square brackets) arranged bi-serially and ovicells (arrowed). (**c**) One live ancestrula (a), the first feeding zooid developed from a larva, and a juvenile *B. neritina* colony (j) with two fully developed autozooids with extended lophophores (l), at the base of which are the mouths of each feeding zooid.

Colloquially referred to as “moss animals”, these nearly microscopic colonial animals with lattice-like connections compose the phylum Bryozoa (Fig. 1). The bryozoans can live in fresh and salt water, mostly in shallow depths less than 100 meters. As Protostomes, bryozoans have a deep evolutionary past^1^. Bryozoan or bryozoan-like fossils have been dated to at least 470 MYA and possibly 550 MYA in the Ediacaran^2^. The long evolution history may explain the extensive radiation to over 5000–6000 estimated, mostly marine, bryozoan species^3^, though other researchers count about 4500 ectoprocta species^4,5^.

Bryozoans were previously classified as the phylum Ectoprocta^4^. However, the phylogenetic placement of bryozoans remains uncertain^6^. Genome sequences could assist phylogenetic analyses, possibly by providing new markers for study^7^. To date, no complete ectoprocta or bryozoan nuclear genomes appear conceptually nor have been completed^8^.

In the 1960s, *Bugula neritina* was found to possess a group of macrolide polyketide lactones called the bryostatins, which are promising anti-neoplastic agents with several modes of action that are important in biomedical research^9,10^. Several studies have shown that bryostatins originate from a bryozoan bacterial symbiont “Candidatus Endobugula sertula”^11–13^. Sequencing and understanding the genome of this species as a representative of a little known phylum may reveal novel mechanisms for how useful natural products can be generated and the extent of host-microbe interactions. This effort adds to the growing catalogue of marine invertebrate genomes supported by the Global Invertebrate Genomics Alliance^14^.

To fill in a gap in the sequencing of animal genomes for understanding the tree of life, we sequenced and assembled the first nuclear Bryozoan genome - the draft genome of *B. neritina* - using PacBio and Illumina HiSeq data.

We assembled a draft genome of 214 Mb with 3,547 contigs and N50 of 94 kb (see Table 1 for details). Overall, the *B.neritina* genome displayed a low to moderate repetitive DNA content - repeats comprise 25.9% of the draft genome (see Supplemental Table 1). We have predicted 25,318 protein-coding genes with functional annotation and assigned orthologs from the eggNOG database^15^.

**Table 1:** Genome assembly statistics.

# contigs (> = 1,000 bp)	3547
# contigs (> = 50,000 bp)	1207
Total length (> = 1,000 bp)	214,69 Mb
Largest contig	1,32 Mb
N50	94,086 bp
L50	595
GC (%)	35.26

**Fig. 2:**
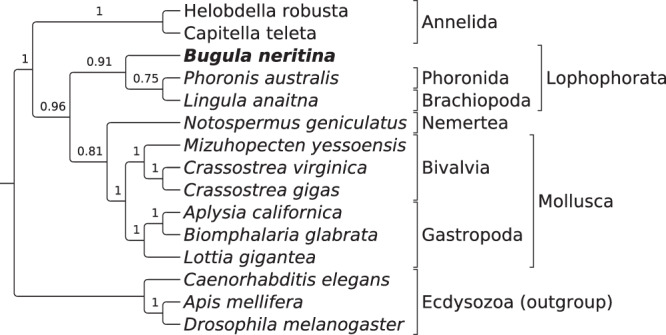
Coalescent species tree of Lophotrochozoa (Spiralia) inferred from 57 BUSCO ML phylogenies. Branch supports measured as local posterior probabilities.

Lastly, we constructed a phylogenetic tree with the single-copy orthologs using BUSCO (Benchmarking Universal Single-Copy Orthologs) phylogenomic approach^16^ (Fig. 2). We used available genomes from Sprialia (Lophotrochozoa), and three Ecdysozoa genomes as an outgroup. Only high-quality assemblies, with >80% assembled BUSCOs, were included in the study.

Despite the strong support of the monophyletic origin of Spiralia, the exact phylogenetic relationships inside the group are still not fully determined. The reconstructed tree is generally in agreement with the phylogeny of Spiralia suggested recently by Marlétaz *et al*.^17^ based on transcriptomics analysis. Our analysis supports the monophyly of Lophophorata clade (brachiopods, phoronids and ectoprocts, including *B.neritina*). We cannot strongly support or reject the two other main clades from this work - Tetraneuralia (mollusks and entoprocts) and the clade combining annelids, nemerteans, and platyhelminthes - because of lack of assembled genomes and low posterior probabilities. Our data does not support the inclusion of annelids and nemerteans in a single monophyletic clade, but more data is needed for such a strong statement.

From an evolutionary standpoint, this first bryozoan genome fills a conspicuous gap in the metazoan tree of finished genomes, which currently shows a taxonomic bias due to sampling constraints and accessibility, as well as technology^18^. We also expect sequences that may be related to allorecognition^19^, and some sequences appeared in our analyses with weak similarities to previously identified allorecognition genes (Alr1). The *B. neritina* genome also fits into ambitious initiatives such as the Earth Biogenome Project, which aims to sequence the majority of eukaryotic taxa on the planet^20^, and so this genome fulfills the goals of both GIGA and EBP. Unexpected genetic markers and features in the *B. neritina* genome will likely be revealed after careful comparison with novel genomes from other phyla.

## Methods

### Sample collection and sequencing

Two adult colonies of *B. neritina* were collected by hand from floating docks in August 2015 from the public floating docks in Oyster, Virginia, U. S. A. (GPS coordinates 37.288 N, −75.923 W) and immediately preserved in RNAlater and stored at −20^o^C. Both samples were genotyped using the protocol described in Linneman *et al*.^21^, and found to be the “shallow” (S) genotype. Voucher samples of sequenced individuals have been deposited with the Ocean Genome Legacy with the Accession ID S00642 (https://www.northeastern.edu/ogl/cataloghttps://www.northeastern.edu/ogl/catalog).

High throughput DNA sequencing was first performed on an Illumina HiSeq Because scaffolds could not be fully closed, we then further sequenced eight 20 kb insert libraries on the Pacific Biosciences RS-II instrument using P6-C5 SMRT cells at the University of Florida ICBR. After preliminary quality filtering we obtained 8.8 G of raw reads, or x60 (given a preliminary genome size estimate of 135 Mb based on flow cytometry data). Genomic DNA extraction included a polysaccharide removal step. Pre-existing Illumina HiSeq data from symbiont genome-sequencing efforts (BioProject PRJNA322176) were also used for polishing.

### Read quality check

We analysed reads using the SGA PreQC package^22^ (see Supplemental Data 1). Estimated genome size was 221 Mb, and the result showed a high level of heterozygosity (high frequency of variant branches in the k-de Bruijn graph). On 51-kmer plot we observed two-peak distribution, similar to the oyster dataset, also indicating high heterozygosity level. Based on GC%/k-mer coverage plots, we suspected the contamination by another organism, which was removed on the binning step (see Binning and validation subsection below).

### Genome assembly

The genome was assembled from raw PacBio reads using Canu assembler v1.2^23^. Draft CANU assembly was evaluated using QUAST 5.0.0^24^. Final assembly was polished with Illumina reads in a single round using Pilon v. 1.23^25^.

### Binning and validation

To avoid possible contamination (which is quite possible for marine invertebrate genomes), we binned obtained contigs with Metabat2 v.2.12^26^. We obtained seven bins, and assessed their taxonomic origin and completeness with CheckM^27^ and BUSCO (for possible bacterial and eukaryotic contamination, respectively). Also we extracted SSU rRNA and searched for homology in NCBI nr/nt database.

Two largest bins (134 Mb and 79 Mb) were attributed to *B.neritina*. Among other bins we observed two bacteria (80% and 24% completeness by CheckM), two small (<1 Mb) bins of unknown origin, and one uncultured labyrinthulid (15 Mb, 81.2% completeness by BUSCO). After keeping the *B.neritina* bins, the genome size was 214 Mb, close to the k-mer based estimation. Assembled genome was subjected to the contamination screen during the submission to the NCBI Assembly database, and no contamination was detected.

### Repeat annotation and gene prediction

First, we analyzed de novo repetitive sequences using RepeatModeler v2.0^28^. Using the obtained database, and Metazoan repeat database Repbase we identified and masked repeats in the draft genome using RepeatMasker v.4.0.6 (http://www.repeatmasker.org/http://www.repeatmasker.org/). Coding regions were predicted using AUGUSTUS v3.3.1^29^ using previously published transcriptome of B.neritina^30^ as hints. The genes were annotated by eggNOG-mapper^15^.

### Phylogenomic reconstruction

The phylogenomic tree was reconstructed using BUSCO Phylogenomics utility script^31^, with default parameters in the SUPERTREE mode. For the reconstruction we were using all available high-quality Spiralian genomes and three Ecdysozoans as an outgroup. “High quality” was defined as >80% of assembled BUSCOs from the database eukaryota_odb10. Following genomes were included in the final reconstruction: *Helobdella robusta* GCF_000326865.1, *Capitella teleta* GCA_000328365.1, *Phoronis australis* GCA_002633005.1, *Lingula anatina* GCF_001039355.2, *Notospermus geniculatus* GCA_002633025.1, *Mizuhopecten yessoensis* GCF_002113885.1, *Crassostrea virginica* GCF_002022765.2, *C. gigas* GCF_000297895.1, *Aplysia californica* GCF_000002075.1, *Biomphalaria glabrata* GCF_000457365.1, *Lottia gigantea* GCF_000327385.1, *Caenorhabditis elegans* GCF_000002985.6, *Drosophila melanogaster* GCF_000001215.4, *Apis mellifera* GCF_003254395.2.

57 BUSCOs were single copy in all 15 species. Each BUSCO group was aligned with MUSCLE^32^, trimmed with trimAl^33^, and ML phylogeny for each BUSCO was generated using IQ-TREE^34^. Coalescent species tree was inferred with Astral v.5.7.3^35^.

## Data Records

Assembled sequences along with gene annotation, have been deposited at NCBI Assembly database as ASM1079987v2^36^. PacBio raw reads have been deposited to NCBI SRA database as SRR11146886^37^. Illumina raw reads have been deposited to NCBI SRA database as SRP081292^38^ as part of the earlier project to characterize the genome of the uncultured bryostatin-producing endosymbiont “Candidatus Endobugula sertula”. The *B. neritina* draft genome (PRJNA498596) will also be included in the umbrella Global Invertebrate Genomics Alliance (GIGA) whole genome dataset, BioProject PRJNA649812, for aquatic non-vertebrate metazoa.

## Technical Validation

We evaluated the completeness of the genome assembly using Benchmarking Universal Single-Copy Orthologs (BUSCO) v2.0^16^. This method relies on a defined set of ultra-conserved eukaryotic protein families for building a highly reliable set of gene annotations. The results showed that 86.3% (220 out of 255 BUSCOs) of the Eukaryota dataset were identified as complete in the *B. neritina* assembly (see Table 2). Together, the results indicated that our dataset represented a genome assembly with a high level of coverage. We also evaluated the quality of the assembly in terms of gene content using the Core Eukaryotic Genes Mapping Approach (CEGMA) pipeline^39^. We used a set of 248 core ultra-conserved genes, and in our analyses 96.19% of these genes were detected. The gene space completeness statistics showed that the assembly can be used for annotation and subsequent analysis.

**Table 2:** BUSCO assessment of the *B.neritina* genome assembly.

Total BUSCO groups searched	255
Missing BUSCOs (M)	26
Fragmented BUSCOs (F)	9
Complete and duplicated BUSCOs (D)	14
Complete and single-copy BUSCOs (S)	206
Complete BUSCOs (C)	220

C:86.3%[S:80.8%, D:5.5%], F:3.5%, M:10.2%, n:255.

## Supplementary information

Supplementary Information

## Data Availability

The execution of this work was not involved using any custom code.
