# Supplementary material for: Draft genome of Bugula neritina, a colonial animal packing powerful symbionts and potential medicines
Source: Sci Data. 2020 Oct 20;7:356. doi: 10.1038/s41597-020-00684-y (PMC7576161; doi:10.1038/s41597-020-00684-y)
Supplement: Supplementary file 1 — Supplementary Information [file 41597_2020_684_MOESM1_ESM.pdf]

Table of contents

Supplemental Data 1 (PreQC results)................................2
Supplemental Table 1 (Repeat content)..............................6

Supplemental Data 1. SGA Preqc Results.

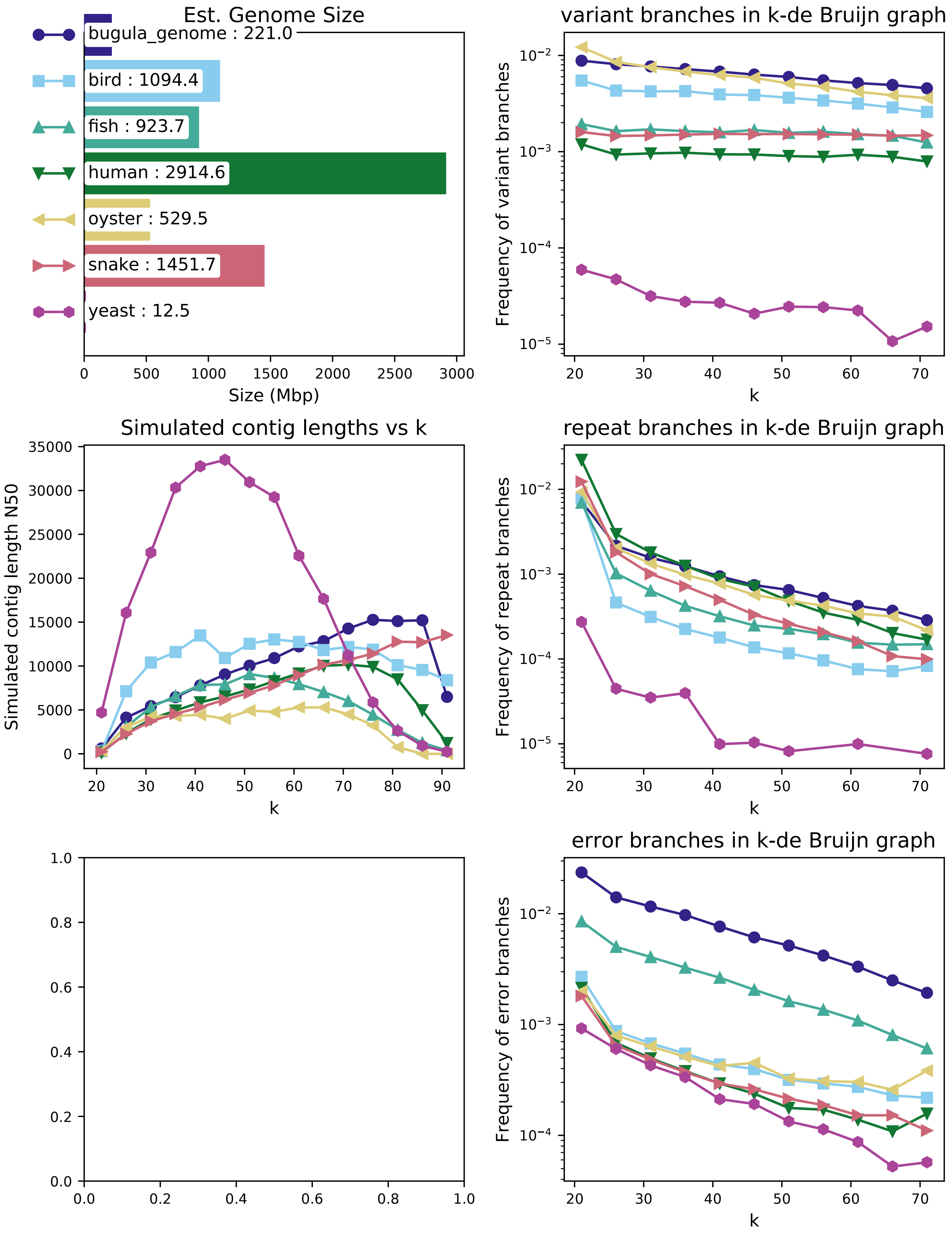

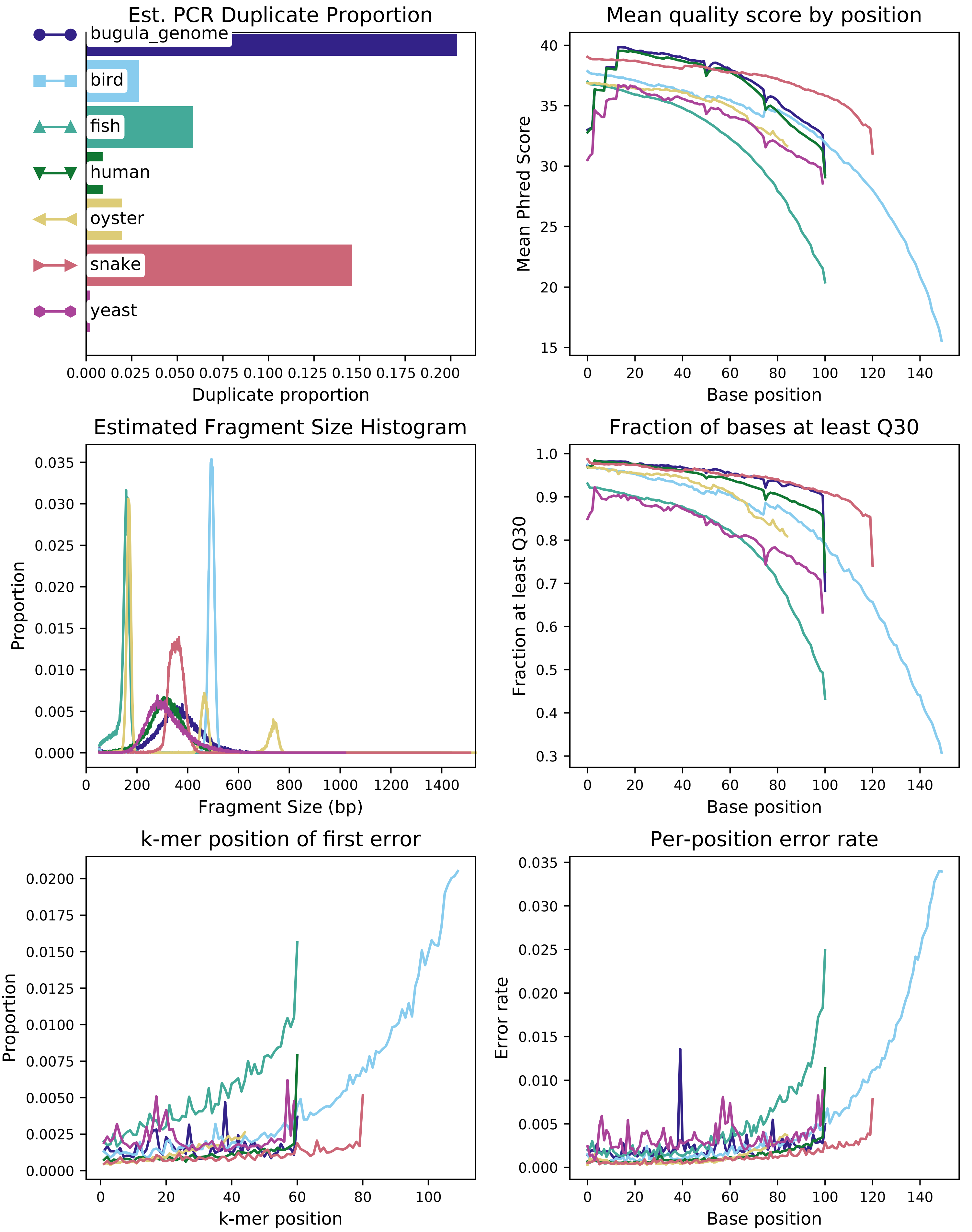
Est. PCR Duplicate Proportion
bugula_genome
bird
fish
human
oyster
snake
yeast
0.000 0.025 0.050 0.075 0.100 0.125 0.150 0.175 0.200
Duplicate proportion
Mean quality score by position
Mean Phred Score
15 20 25 30 35 40
0 20 40 60 80 100 120 140
Base position
Estimated Fragment Size Histogram
Proportion
0.000 0.005 0.010 0.015 0.020 0.025 0.030 0.035
0 200 400 600 800 1000 1200 1400
Fragment Size (bp)
Fraction of bases at least Q30
Fraction at least Q30
0.3 0.4 0.5 0.6 0.7 0.8 0.9 1.0
0 20 40 60 80 100 120 140
Base position
k-mer position of first error
Proportion
0.0000 0.0025 0.0050 0.0075 0.0100 0.0125 0.0150 0.0175 0.0200
0 20 40 60 80 100
k-mer position
Per-position error rate
Error rate
0.000 0.005 0.010 0.015 0.020 0.025 0.030 0.035
0 20 40 60 80 100 120 140
Base position

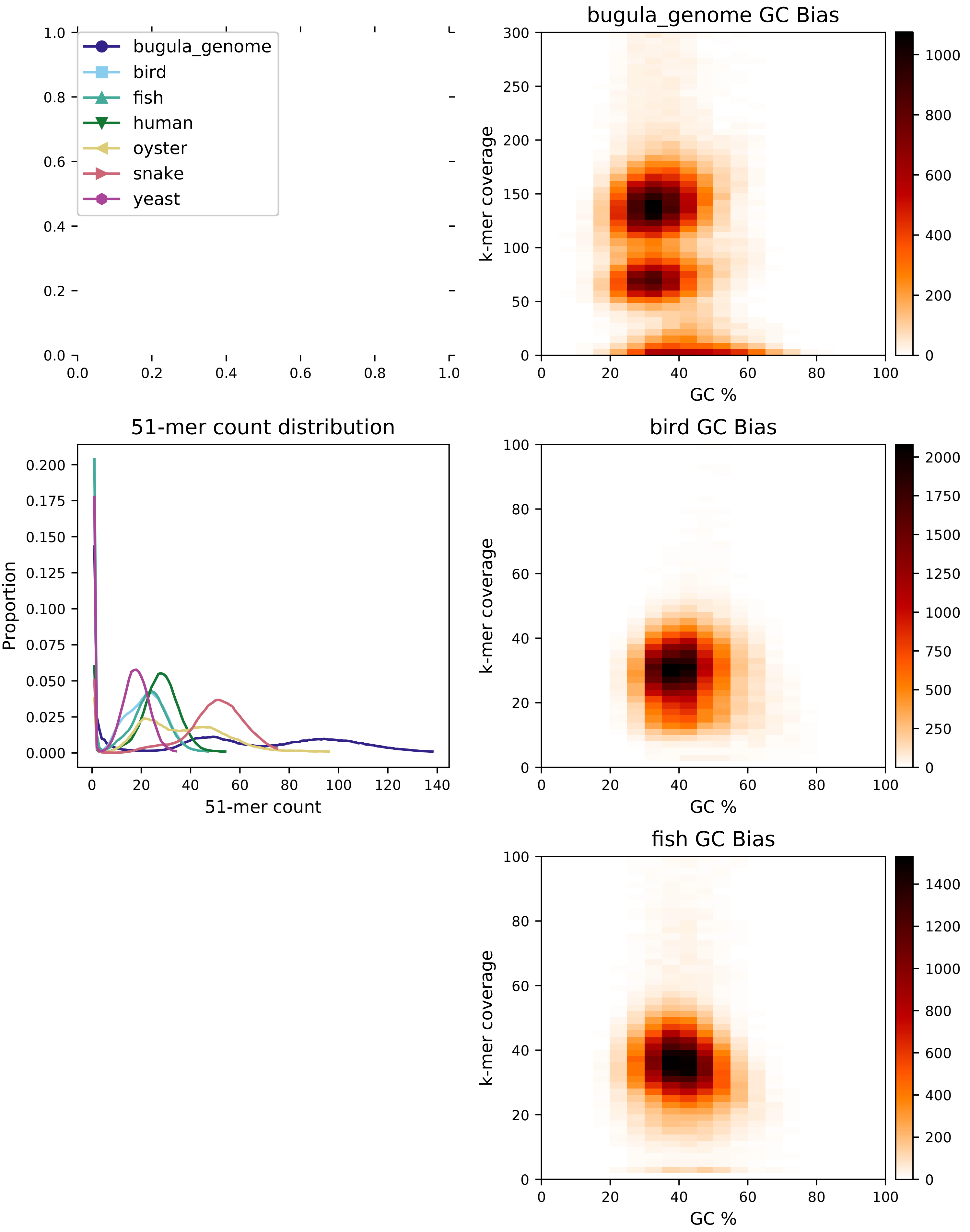
bugula_genome
bird
fish
human
oyster
snake
yeast
bugula_genome GC Bias
k-mer coverage
GC %
51-mer count distribution
Proportion
51-mer count
bird GC Bias
k-mer coverage
GC %
fish GC Bias
k-mer coverage
GC %

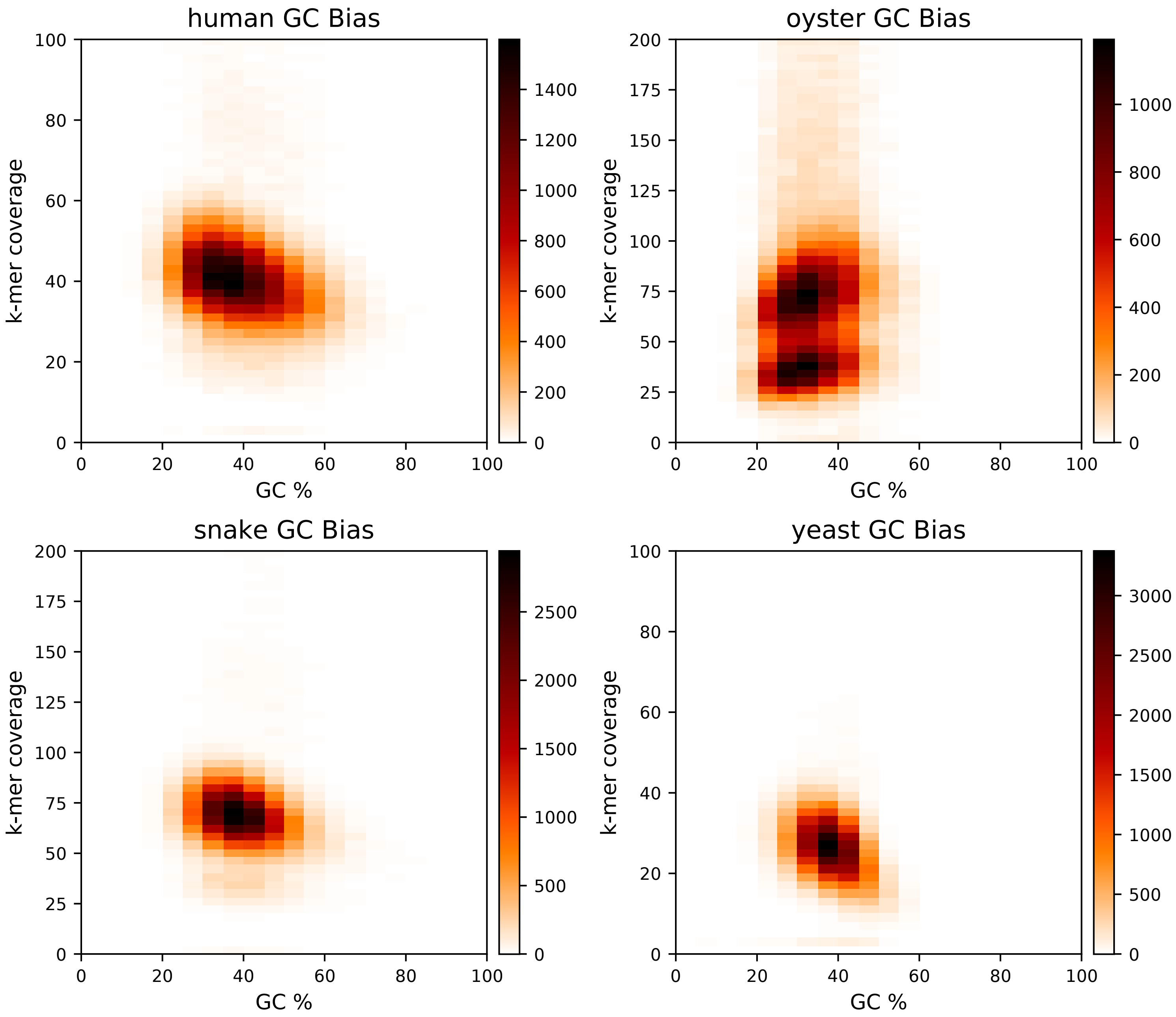
human GC Bias
oyster GC Bias
snake GC Bias
yeast GC Bias
k-mer coverage
GC %

Supplemental Table 1. Repeat content of the *B.neritina* genome

```
==================================================
sequences:          3547
total length:  214693723 bp  (214693723 bp excl N/X-runs)
GC level:         34.72 %
bases masked:   55542846 bp ( 25.87 %)
==================================================
               number of      length   percentage
               elements*    occupied  of sequence
--------------------------------------------------
Retroelements        27447     18300120 bp    8.52 %
   SINEs:               46         2112 bp    0.00 %
   Penelope           2397       785481 bp    0.37 %
   LINEs:            14371      4770421 bp    2.22 %
    CRE/SLACS            0            0 bp    0.00 %
     L2/CR1/Rex       3895      1796007 bp    0.84 %
     R1/LOA/Jockey       0            0 bp    0.00 %
     R2/R4/NeSL        793       382779 bp    0.18 %
     RTE/Bov-B        2100      1154705 bp    0.54 %
     L1/CIN4          5186       651449 bp    0.30 %
   LTR elements:     13030     13527587 bp    6.30 %
     BEL/Pao          1006       758652 bp    0.35 %
     Ty1/Copia         336       311928 bp    0.15 %
     Gypsy/DIRS1      9542     12168188 bp    5.67 %
       Retroviral      329        83829 bp    0.04 %

DNA transposons      28516      7942958 bp    3.70 %
   hobo-Activator     2880       662976 bp    0.31 %
   Tc1-IS630-Pogo     1570       565921 bp    0.26 %
   En-Spm                0            0 bp    0.00 %
   MuDR-IS905            0            0 bp    0.00 %
   PiggyBac              0            0 bp    0.00 %
   Tourist/Harbinger     0            0 bp    0.00 %
   Other (Mirage,       58        27429 bp    0.01 %
    P-element, Transib)

Rolling-circles          0            0 bp    0.00 %
Unclassified:        84448     21541525 bp   10.03 %
Total interspersed repeats:    47784603 bp   22.26 %
Small RNA:           11631      1882093 bp    0.88 %
Satellites:              0            0 bp    0.00 %
Simple repeats:      68160      5532140 bp    2.58 %
Low complexity:       6521       344010 bp    0.16 %
==================================================
```

```
* most repeats fragmented by insertions or deletions
  have been counted as one element.
  Runs of >=20 X/Ns in query were excluded in % calcs.
  RepeatMasker Combined Database: Dfam_3.1
```
